# Activation of NLRP3 inflammasome in lung epithelial cells triggers radiation-induced lung injury

**DOI:** 10.1186/s12931-023-02331-7

**Published:** 2023-01-24

**Authors:** Xinrui Rao, Dong Zhou, Huilin Deng, Yunshang Chen, Jian Wang, Xiaoshu Zhou, Xiaohua Jie, Yingzhuo Xu, Zilong Wu, Geng Wang, Xiaorong Dong, Sheng Zhang, Rui Meng, Chuangyan Wu, Shijie Xing, Kai Fan, Gang Wu, Rui Zhou

**Affiliations:** 1 Cancer Center, Union Hospital, Tongji Medical College, Huazhong University of Science and Technology, Wuhan, 430022 China; 2 Institute of Radiation Oncology, Union Hospital, Tongji Medical College, Huazhong University of Science and Technology, Wuhan, 430022 China; 3 Department of Gastrointestinal Surgery, Union Hospital, Tongji Medical College, Huazhong University of Science and Technology, Wuhan, 430022 China; 4 Department of Thoracic Surgery, Union Hospital, Tongji Medical College, Huazhong University of Science and Technology, Wuhan, 430022 China

**Keywords:** Radiation-induced lung injury, Lung fibrosis, Inflammasomes, NLRP3, Glucose metabolism

## Abstract

**Background:**

Radiation-induced lung injury (RILI) is the most common and serious complication of chest radiotherapy. However, reported radioprotective agents usually lead to radiation resistance in tumor cells. The key to solving this problem is to distinguish between the response of tumor cells and normal lung epithelial cells to radiation damage.

**Methods:**

RNA-Seq was used to recognize potential target of alleviating the progression of RILI as well as inhibiting tumor growth. The activation of NLRP3 inflammasome in lung epithelial cells was screened by qRT-PCR, western blotting, immunofluorescence, and ELISA. An in vivo model of RILI and in vitro conditioned culture model were constructed to evaluate the effect of NLRP3/interleukin-1β on fibroblasts activation. ROS, ATP, and (NADP)^+^/NADP(H) level in lung epithelial cells was detected to explore the mechanism of NLRP3 inflammasome activation. The lung macrophages of the mice were deleted to evaluate the role of lung epithelial cells in RILI. Moreover, primary cells were extracted to validate the results obtained from cell lines.

**Results:**

NLRP3 activation in epithelial cells after radiation depends on glycolysis-related reactive oxygen species accumulation. DPYSL4 is activated and acts as a negative regulator of this process. The NLRP3 inflammasome triggers interleukin-1β secretion, which directly affects fibroblast activation, proliferation, and migration, eventually leading to lung fibrosis.

**Conclusions:**

Our study suggests that NLRP3 inflammasome activation in lung epithelial cells is essential for radiation-induced lung injury. These data strongly indicate that targeting NLRP3 may be effective in reducing radiation-induced lung injury in clinical settings.

**Supplementary Information:**

The online version contains supplementary material available at 10.1186/s12931-023-02331-7.

## Introduction

Chest radiotherapy is critical in patients with thoracic and breast malignancies. However, radiation-induced damage to the lungs remains an important barrier to better implementation of radiotherapy. Radiation-induced lung injury (RILI) can be divided into early radiation pneumonitis (RP) and late radiation-induced pulmonary fibrosis (RPF) [1]. RILI is associated with an increased risk of death, disability, and a decline in quality of life [2]. RPF is characterized by fibroblast proliferation, collagen deposition, and destruction of normal lung architecture, resulting in dyspnea and respiratory failure [3]. Given the increasing number of long-term cancer survivors, it’s of vital importance to mitigate or prevent late effects of radiotherapy. However, the biology and molecular mechanisms of RPF have not been fully elucidated.

Inflammation is a key element of RILI. Inflammasomes are essential for innate immunity and responses to cellular damage. The activation of inflammasomes leads to inflammatory death called pyroptosis [4]. The nucleotide-binding oligomerization domain-like receptor family pyrin domain-containing (NLRP)3 inflammasome is the most widely studied inflammasome and can be activated by an array of stimuli, and has been linked to the pathogenesis of several inflammatory disorders, including cryopyrin-associated periodic syndromes, Alzheimer’s disease, gout, autoinflammatory diseases, and atherosclerosis [5]. Previous studies have also shown that NLRP3 inflammasome is involved in fibrotic diseases, including idiopathic pulmonary fibrosis (IPF) [6]. NLRP3 interacts with a bipartite adaptor protein, known as an apoptosis-associated speck-like protein containing a caspase-recruitment domain (ASC), and promotes the recruitment of pro-caspase-1 (CASP1) to the inflammasome complex. Active CASP1 then cleaves the cytokines pro-interleukin (pro-IL)-1β and pro-IL-18 into mature and biologically active forms [7]. Cytokine interleukin (IL)-1β is a potent pro-inflammatory cytokine that has been proven to promote collagen synthesis [8]. IL-1β has been shown to play a role in the occurrence, invasion, and metastasis of multiple kinds of tumors [9]. Whether inhibiting the NLRP3 inflammasome has the potential to mitigate radiation damage to normal lung tissue and to inhibit further progression of tumors remains to be explored.

Some studies have suggested the involvement of inflammasomes in RILI, but they have mainly focused on macrophages [10–12]. The role of lung epithelial cells in RILI remains to be elucidated. Airway epithelial cells are considered the host’s first line of defense against harmful invasion and participate in the initiation and progression of inflammation. Airway epithelial cells express a large number of pattern recognition receptors, which can quickly perceive pathogen-associated molecular patterns as well as damage-associated molecular patterns released by damaged tissue, and then make a series of responses, such as the release of cytokines and chemokines [13]. In asthma, airway epithelial cells sense allergens and recruit immune cells. Recruited immune cells secrete a large number of chemokines and cytokines that cause further damage to epithelial cells, resulting in the enhancement and persistence of the inflammatory response [14]. Therefore, studying the role of inflammasomes in the airway epithelium in RILI may provide new insights for the prevention of epithelial dysfunction.

Metabolic alterations are increasingly being recognized as important pathogenic processes that underlie fibrotic diseases [15]. Glucose metabolism begins with its conversion into pyruvate and ends with lactic acid production in the cytoplasm, termed glycolysis [16]. The metabolic shift that cancer cells undergo towards aerobic glycolysis, rather than being fully metabolized to carbon dioxide via mitochondrial oxidative phosphorylation (OXPHOS), was identified as the Warburg effect [17]. The Warburg effect results in quicker production of biosynthetic intermediates, as well as adenosine 5′-triphosphate (ATP), which can then be used for protein synthesis and cell proliferation. Similarly, enhanced protein synthesis and production of the same biosynthetic intermediates are hallmark of fibrosis [18]. In addition, the Warburg effect leads to IPF by enhancing myofibroblast activation [19]. Therefore, targeting the Warburg effect may be a potential therapeutic strategy for fibrosis. However, its role in RPF remains unclear.

Here, we aimed to differentiate the radiation response between lung tumor cells and normal lung epithelial cells, and investigated the function of NLRP3 inflammasomes in lung epithelial cells in RILI. Notably, we preliminarily revealed the relationship between glucose metabolism and inflammasome activation in RILI. These data provide evidence that NLRP3 may serve as a promising therapeutic target in RILI.

## Methods and materials

### Cell isolation and culture

The human bronchial epithelial cell line BEAS-2B and HBE, non-small cell lung cancer cell line A549, and small cell lung cancer cell line H446 were purchased from the Cell Bank of the Chinese Academy of Sciences (Beijing, China) and they were cultured in RPMI-1640 supplemented with 10% fetal bovine serum (FBS) at 37 °C in 5% carbon dioxide._._ Primary human bronchial epithelial cell (PHBE) cells and primary lung fibroblasts (HLF) were isolated and cultured from freshly surgically removed human lung tissue as previously described [20]. Primary mouse embryo fibroblasts (MEF) were isolated from embryos of pregnant C57/BL6 mouse. The uterus was removed and placed in balanced phosphate solution. After removing the head and other internal organs, the embryos were cut into small pieces. The tissue was centrifugated at 1000 rpm/5 min and resuspended the in trypsin, and then was digested in a 37 °C incubator for approximately 30 min. The cells were centrifuged at 1000 rpm for 5 min and cultured in RPMI-DMEM containing 10% FBS.

### Human lung specimens

Fibrotic lung specimens were obtained from 8 patients with organizing pneumonia who underwent partial lung resection. Control lung tissue was obtained from 11 para-cancerous tissues of patients with lung cancer. The use of these samples was approved by the ethics review board.

### Irradiation of cells

The cells were irradiated with X-ray radiation by using the Varian Vital Beam Linear Accelerator (Varian Medical Systems) (dose rate of 4 Gy/min, source–surface distance of 100 cm). The selection of radiation dose was based on our previous studies and other publications [21–23].

### Quantification of cytokines

Bronchoalveolar lavage fluid (BALF) and serum samples of mice were collected and centrifuged. The supernatants were used to quantify IL-1β and IL-18 levels. IL-1β and IL-18 levels in the culture supernatants of cells, BALF, or serum of mice were measured by enzyme-linked immunosorbent assay (ELISA) (Proteintech) according to the manufacturer’s instructions.

### Quantitative reverse transcription-polymerase chain reaction (qRT-PCR)

Total ribonucleic acid (RNA) was extracted from the cells using TRIzol reagent (Invitrogen) according to standard procedures. qRT-PCR was performed using SYBR Premix Ex Taq reagents (TaKaRa) in two steps according to the manufacturer’s instructions. Primer sequences used are listed in Additional file 2: Table S1.

### Intracellular reactive oxygen species (ROS), ATP and nicotinamide adenine dinucleotide phosphate (NADP)^+^/NADP(H) measurement

Intracellular ROS were detected using the fluorescent probe DCFH-DA (Beyotime). Briefly, lung epithelial cells were stained with DCFH-DA for 30 min, harvested, centrifuged, and washed with phosphate-buffered saline (PBS). The stained cells (approximately 10^5^ cells/sample) were analyzed by flow cytometry using a FACSCalibur system. Intracellular ATP and NADP+/NADPH were detected using ATP Assay Kit (Beyotime) and NADP+/NADPH Assay Kit with WST-8 (Beyotime), respectively, according to the manufacturer’s instructions.

### Measurement of lactic acid secretion and glucose uptake

Culture supernatants of irradiated lung epithelial cells were collected at different time points, and lactic acid and glucose levels were measured using a lactic acid assay kit (Nanjing Jiancheng) and Glucose Assay Kit (Nanjing Jiancheng), respectively, according to the manufacturer’s instructions.

### EdU essay

The EdU Cell Proliferation Kit (Beyotime) was used to detect the proliferation ability of fibroblasts, following the manufacturer’s instructions. Briefly, 24 h after IL-1β treatment, EdU was added to the cells and incubated for 2 h, after which the cells were fixed and washed again. Following permeabilization, the click additive solution was added to the cells and incubated for 30 min. Finally, cells were stained with Hoechst 33,342 for 10 min, observed, and photographed under a microscope.

### Western blotting

Western blot analysis was performed according to standard procedures, as previously described [21]. The following primary antibodies were used: anti-NLRP3 (1:1000, Abcam), anti-ASC (1:1000; Proteintech), anti-GSDMD (1:1000; Abcam), anti-CASP1 (1:1000, Proteintech), anti-DPYSL4 (1:1000; Abcam), anti-p53 (1:1000; Santa Cruz), and anti-β-actin (1:5000; Proteintech). Following incubation with the primary antibody, the membranes were washed and probed with secondary antibodies (1:5000; Proteintech) for 1 h. Proteins were visualized by chemiluminescence.

### Silencing of NLRP3, DPYSL4 and IL-1β by small interfering RNA (siRNA)

Transfection of NLRP3, DPYSL4, or IL-1β siRNA and negative control siRNA was performed using Lipofectamine RNAiMAX (Invitrogen) according to the manufacturer’s instructions. Transfection efficacy was detected by qRT-PCR and western blotting 48 h post transfection. The siRNA sequences were as follows:

- Scramble siRNA, 5′-UUCUCCGAACGUGUCACGU-3′
- SiNLRP3, 5′-GCUUCAGGUGUUGGAAUUA-3′
- SiIL-1β, 5′-CGAUUUGUCUUCAACAAGA-3′
- SiDPYSL4, 5′-CGGUUCACACUACUGGAGCAA-3′

### Histopathology and histochemistry

Serial paraffin sections of lung tissue were stained with Masson trichrome stain for analysis of pathological changes in the airway. Immunohistochemical (IHC) staining was performed as previously described [24]. NLRP3 primary antibody (1:50; Novus) and DPYSL4 primary antibody (1:200; Abcam) was used. The IHC score was categorized as negative (1), weakly positive (2), moderately positive (3), and strongly positive (4).

### Immunofluorescence

Approximately 2 × 10^4^ BEAS-2B cells were seeded in 24 well plates coated with sterile glass coverslips. The next day the cells were given 10 Gy X-ray radiation, then the cells were fixed with 4% paraformaldehyde 24 h later. The cells were permeabilized with PBS containing 0.2% Triton X-100 for 15 min. Next, the cells were blocked with 5% bovine serum albumin and incubated with primary antibodies (NLRP3: NOVUS, 1:100; ASC: Proteintech, 1:100) at 4 °C overnight. The next day, the cells were washed and incubated with secondary antibodies. The cells were then stained with DAPI, observed, and photographed under a microscope.

### Plate colony formation assays

Lung cancer cells treated with MCC950 or PBS for 24 h were seeded in six-well plates (200 cells per well). After 2 weeks, the cells were fixed and stained with crystal violet, and colonies that consisted of at least 50 cells were counted.

### In vivo experiments

C57BL/6 mice (6 weeks old, female) were raised at the standard specific pathogen free animal housing. IL-1 receptor (IL-1R) knockout mice were kindly provided by professor Feng Shao (National Institute of Biological Sciences, Beijing, China). All animal procedures were performed in accordance with the National Institutes of Health Guide for the Care and Use of Laboratory Animals.

To establish the RILI mouse model, mice were subjected to a single 16 Gy thoracic X-ray irradiation. Mice were sacrificed at different time points. Lung tissue, BALF, and serum were collected for further examination.

To deplete the lung macrophages of the mice, clodronate liposomes (FomuMax) were administered to C57BL/6 mice. Briefly, the mice received both intranasal and tail vein administration of the first dose, and then clodronate liposomes were administered through the tail vein every 2 days. The depletion efficiency was confirmed by flow cytometry.

For NLRP3 inhibition, the mice were given intraperitoneal injection of MCC950 (10 mg/kg) 24 h after 16 Gy thoracic radiation and continued for 2 months with injections every other day. Mice were sacrificed 5 months after radiation.

### Genotyping of mice

Mouse tail deoxyribonucleic acid (DNA) was extracted using Quick Genotyping Assay Kit for Mouse Tails (Beyotime). Polymerase chain reaction (PCR) and gel electrophoresis were performed to detect the genotypes of the mice. The primer sequences used are listed in Additional file 2: Table S2.

### ROS measurement of mouse lung tissue

The levels of ROS in lung tissues of mouse were measured with Reactive Oxygen Species Assay Kit (Applygen) according to the manufacture’s instruction. The single lung cell suspensions were incubated with the DHE fluorescent probe for 30 min at the final concentration of 10 µM. Then the cells were washed with PBS and the fluorescence intensity was measured.

### RNA sequencing

Cells were lysed with TRIzol reagent and sent for transcriptome sequencing in liquid nitrogen (BGI, Shenzhen, China). Total RNA was extracted from cells. Oligo(dT)-attached magnetic beads were used to purify the mRNA. Single-stranded circular DNA was used as the final library. The final library was amplified with phi29 to form a DNA nanoball (DNB), which had more than 300 copies of one molecule. DNBs were loaded into the patterned nanoarray and single end 50 base reads were generated on the BGIseq500 platform (BGI; Shenzhen, China). The original RNA-seq data generated in the study has been uploaded to the Gene Expression Omnibus under registration number GSE211118.

Differential expression analysis was performed with a Q-value of < 0.05. In addition, functional enrichment analysis, including KEGG pathways, Gene Ontology [25] enrichment, and gene set enrichment analysis (GSEA), was performed.

### Statistical analysis

Experimental data are expressed as the mean ± standard error of the mean. Statistical differences among groups were assessed using SPSS 26.0 (IBM Corp; Armonk, NY). In all experiments, categorical variables between groups were compared using the χ^2^ test, and continuous variables were analyzed using Student’s t-test (two-tailed) or analysis of variance, as appropriate. All data met the assumptions of the tests, and statistical tests were justified, as appropriate. Differences were considered statistically significant at P < 0.05 (*P < 0.05, **P < 0.01, ***P < 0.001, and ****P < 0.0001). All experiments were repeated at least three times.

## Results

### The different response pattern between lung epithelial cells and lung cancer cells after radiation revealed by RNA-sequencing (RNA-seq)

**Fig. 1:**
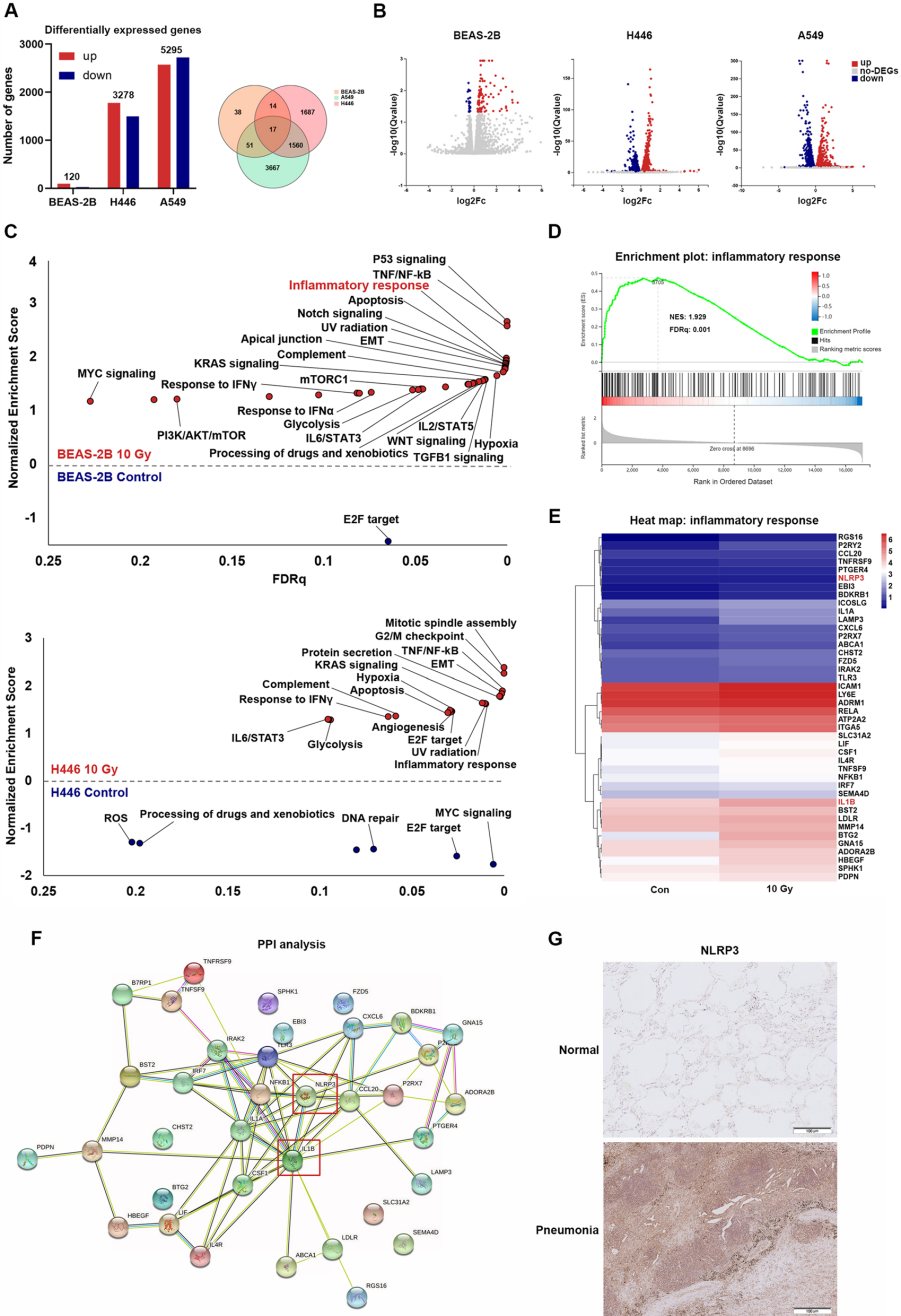
The different response pattern between lung epithelial cells and lung cancer cells after radiation. **A** Number of differentially expressed genes in BEAS-2B, H446 and A549 after radiation (n = 3). **B** The volcano plot shows the differentially expressed genes in BEAS-2B, H446 and A549 (n = 3). **C** GSEA analysis of signaling pathways affected by radiation in BEAS-2B and H446 (n = 3). **D** GSEA plots showing the enrichment of the inflammatory response pathway in the irradiated BEAS-2B (n = 3). **E** Heatmap showing the change of core genes enriched in the inflammatory response pathway (n = 3). **F** PPI analysis showing that the NLRP3/IL-1β axis was at the center of the top 44 genes enriched in the inflammatory response pathway (n = 3). **G** Representative images of IHC staining for NLRP3 in serial sections of organizing pneumonia and normal lung tissue. Scale bar, 100 μm. *GSEA* gene set enrichment analysis; *PPI* protein–protein interaction, *IL* interleukin, *IHC* immunohistochemistry

To determine whether ionizing radiation has different biological effects on normal lung cells and cancer cells, we treated the lung epithelial cell line BEAS-2B, non-small cell lung cancer cell line A549, and small cell lung cancer cell line H446 with 10 Gy X-ray radiation, and RNA-seq was performed. The results showed 120 differentially expressed genes in BEAS-2B cells, 3278 differentially expressed genes in H446 cells, and 5295 differentially expressed genes in A549 cells, with only 17 of these genes commonly expressed in all cell lines, revealing the potential different response patterns between lung epithelial cells and lung cancer cells after radiation (Fig. 1A, B). GSEA revealed that lung epithelial cells strongly expressed genes involved in the inflammatory response and apoptosis pathways, while lung cancer cells highly upregulated genes associated with DNA damage repair (Fig. 1C, D). We further analyzed the top 44 genes enriched in inflammatory response pathway in irradiated lung epithelial cells, and protein–protein interaction analysis showed that the NLRP3/IL-1β axis was at the center (Fig. 1E, F). To determine whether the NLRP3 inflammasome is associated with pulmonary fibrosis, the expression of NLRP3 was analyzed in serial sections of organizing pneumonia and normal lung tissue from the patients. We found that NLRP3 was overexpressed in organizing pneumonia tissues compared to normal lung tissues (Fig. 1G). These results indicate a correlation between NLRP3/IL-1β activation and inflammation post-radiation.

### Radiation activates NLRP3 inflammasome canonical pathways in lung epithelial cells

**Fig. 2:**
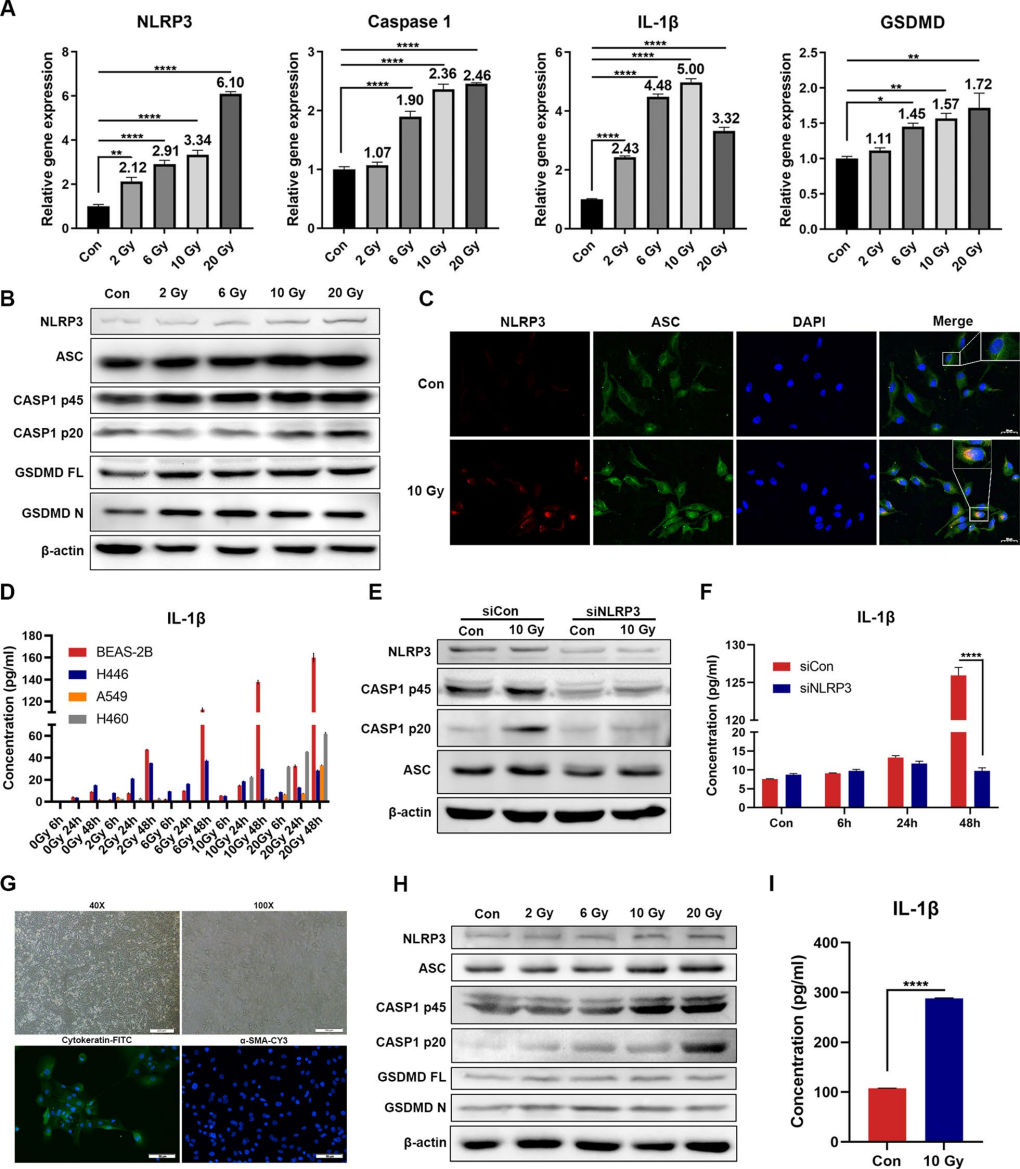
NLRP3 inflammasome is activated and induces pyroptosis after radiation of lung epithelial cells. **A** qRT-PCR showing that the mRNA level of NLRP3, CASP1, IL-1β and GSDMD expression were increased after radiation in BEAS-2B (n = 3). **B** Western blotting showing that the protein level of NLRP3, CASP1 cleavage and GSDMD cleavage were increased after radiation in BEAS-2B (n = 3). **C** Representative fluorescent microscopy images of BEAS-2B that was co-stained for NLRP3, ASC and DAPI before and after radiation (n = 3). Scale bar, 50 μm. **D** ELISA results of IL-1β in supernatant of BEAS-2B, H446, A549 and H460 at different time points after radiation (n = 3). **E** BEAS-2B cells were transfected with the control or siNLRP3 siRNA, and given 10 Gy X-ray radiation, then samples were collected and changes in essential components of NLRP3 inflammasome were detected (n = 3). **F** ELISA results of IL-1β in supernatant of BEAS-2B transfected with control or NLRP3 siRNA at different time point after radiation (n = 3). **G** Microscopy images of PHBE and fluorescent microscopy images of PHBE stained for epithelium-specific cytokeratins and fibroblast marker α-SMA (n = 3). **H** Western blotting showing the level of NLRP3 protein expression, CASP1 cleavage and GSDMD cleavage were increased after radiation in PHBE (n = 3). **I** ELISA results of IL-1β in supernatant of PHBE 48 h after radiation (n = 3). Bar graphs show the means ± SEM; *P < 0.05, **P < 0.01, ***P < 0.001, ****P < 0.0001. *qRT-PCR* quantitative reverse transcription polymerase chain reaction, *ASC* apoptosis-associated speck-like protein containing a caspase-recruitment domain, *IL* interleukin, *ELISA* enzyme-linked immunosorbent assay, *PHBE* primary human bronchial epithelial cells, *SEM* standard error of mean

To estimate the changes in the levels of NLRP3 inflammasome proteins after radiation, qRT-PCR and western blotting analyses of NLRP3 inflammasome canonical pathway proteins were performed. We found that the mRNA levels of NLRP3 inflammasome components, including NLRP3, CASP1 and IL-1β, as well as the pyroptosis marker gasdermin D (GSDMD), were significantly upregulated after radiation in lung epithelial cells, and that radiation increased the protein expression levels of NLRP3, ASC, cleaved CASP1, and cleaved GSDMD in a radiation dose-dependent manner, while NLRP3 expression was not upregulated in lung cancer cells (Fig. 2A, B, Additional file 1: Fig. S1). NLRP3 interacts with ASC to initiate inflammasome assembly. We found that radiation also induced the colocalization of NLRP3 with ASC (Fig. 2C). The IL-1β levels in the supernatants of irradiated BEAS-2B, H446, A549, and H460 cells at different time points were detected by ELISA, and the results showed that irradiated BEAS-2B cells secreted significantly higher amounts of IL-1β compared with irradiated lung cancer cells, which was most evident 48 h post radiation. The change in IL-1β levels correlated with the increase in the radiation dose (Fig. 2D). However, the change in IL-18 levels was very modest in lung epithelium compared with lung cancer cells, indicating a more profound role of IL-1β in lung epithelium-induced inflammation (Additional file 1: Fig. S2).

To determine whether the activation of CASP1 is dependent on NLRP3, BEAS-2B cells were transfected with NLRP3-silencing siRNA. Transfection efficiency was analyzed using western blotting and qRT-PCR. NLRP3 inhibited both the activation of CASP1 and secretion of IL-1β secretion (Fig. 2E, F, Additional file 1: Fig. S3). To further investigate the correlation between NLRP3 inflammasome, lung epithelial cells, and RILI, PHBE were isolated from human bronchial segments. As shown in Fig. 2G, PHBE cells had a typical cobblestone appearance and presented positive immunostaining for the epithelial marker cytokeratin and negative immunostaining for the fibroblast marker α-SMA. Consistent with the above findings, the NLRP3 inflammasome was activated in PHBE after radiation, and the secretion of IL-1β was significantly increased (Fig. 2H, I). Taken together, these results indicate that radiation induces NLRP3 inflammasome activation and pyroptosis in the lung epithelial cells.

Since NLRP3 may be the key molecule in RILI as well as in tumorigenesis and progression, we treated A549 and H446 cells with MCC950, a specific small-molecule inhibitor that selectively blocks NLRP3. The result revealed the inhibition of the proliferation ability of both H446 and A549 by MCC950 (Additional file 1: Fig. S4), indicating that the suppression of NLRP3 may inhibit tumor growth and alleviate the progression of RILI.

### Radiation induced NLRP3 inflammasome activation relies on glycolysis related ROS accumulation

**Fig. 3:**
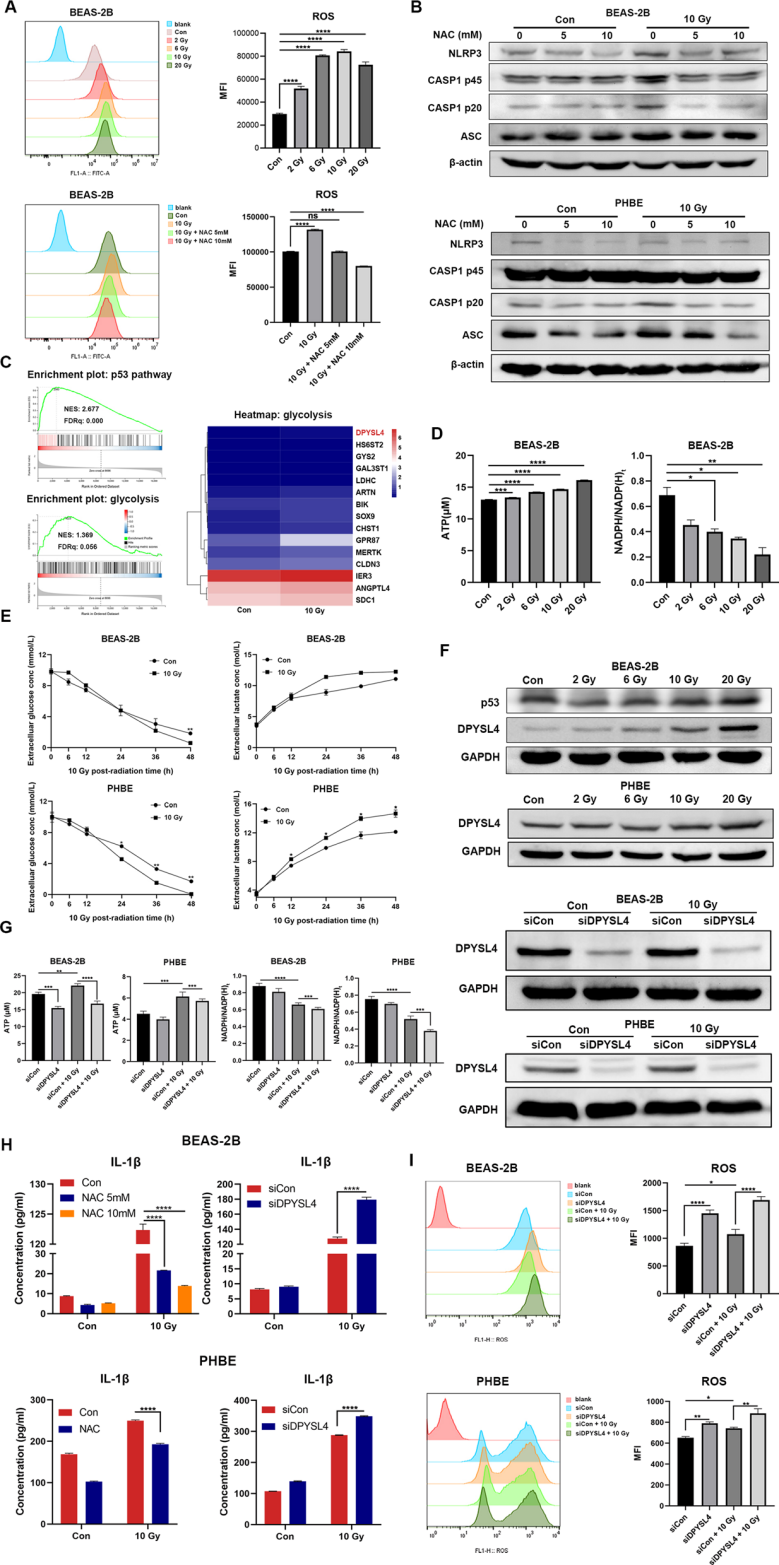
Radiation induced intracellular glucose metabolic change is associated with NLRP3 inflammasome activation. **A** Flow cytometry of the intracellular ROS level at 6 h post radiation pretreated with or without NAC in BEAS-2B (n = 3). **B** Western blotting showing the NLRP3, ASC protein expression, CASP1 cleavage and GSDMD cleavage were inhibited pretreated with NAC after radiation in BEAS-2B and PHBE (n = 3). **C** GSEA plot showing the enrichment of the p53 and glycolysis pathway in the irradiated BEAS-2B, and heatmap of differentially expressed genes in glycolysis pathway (n = 3). **D** Intracellular ATP level was increased and NADPH/NADP(H)_total_ ratio was decreased in the irradiated BEAS-2B (n = 3). **E** Extracellular lactic acid level and extracellular glucose level measured at different time points after radiation in BEAS-2B and PHBE (n = 3). **F** DPYSL4 protein levels were examined by Western blotting in BEAS-2B, and PHBE given different dose of X-ray radiation, or transfected with the control or siDPYSL4 siRNA and analyzed by Western blotting to confirm the knockdown efficiency. **G** Intracellular ATP level and NADPH/NADP(H) total ratio was detected in BEAS-2B and PHBE transfected with the control or siDPYSL4 siRNA  (n = 3). **H** ELISA results of IL-1β in supernatant of BEAS-2B and PHBE 48 h after radiation pretreated with NAC or transfected with control or siDPYSL4 siRNA (n = 3). **I** Flow cytometry of the intracellular ROS level at 6 h post radiation transfected with control or siDPYSL4 siRNA (n = 3). Bar graphs show the mean ± SEM; *P < 0.05, **P < 0.01, ***P < 0.001, ****P < 0.0001 . *MFI* mean fluorescence intensity, *ROS* reactive oxygen species, *NAC* *N*-acetyl-l-cysteine, *ASC* apoptosis-associated speck-like protein containing a caspase-recruitment domain, *GSEA* gene set enrichment analysis, *ATP* adenosine 5′-triphosphate, *NADP* nicotinamide adenine dinucleotide phosphate, *PHBE* primary human bronchial epithelial cells, *siRNA* small interfering ribonucleic acid, *ELISA* enzyme-linked immunosorbent assay, *IL* interleukin, *SEM* standard error of the mean, *ns* not significant

Radiation-induced normal tissue injury is a dynamic and progressive process that begins with ROS production and accumulation [1]. ROS can cause DNA damage, and protein and lipid oxidization, followed by a series of biological processes that may lead to cell death and inflammation [26]. We found that ROS levels were significantly increased in the lung epithelial cell lines BEAS-2B and HBE in a radiation dose-dependent manner (Fig. 3A, Additional file 1: Fig. S5). To investigate whether ROS accumulation in lung epithelial cells triggered NLRP3 inflammasome activation, we used *N*-acetyl-l-cysteine (NAC), to treat lung epithelial cells prior to radiation, and found that NAC largely scavenged intracellular ROS in lung epithelial cells (Fig. 3A, Additional file 1: Fig. S5A). NAC pretreatment abolished radiation-induced NLRP3 inflammasome activation, CASP1 cleavage, and IL-1β secretion in BEAS-2B and PHBE cells, suggesting that ROS accumulation may trigger NLRP3 inflammasome activation in lung epithelial cells (Fig. 3B, H, Additional file 1: Fig. S5B).

According to the RNA-seq results, several pathways involved in metabolic processes, were enriched in irradiated lung epithelial cells (Fig. 3C). To investigate the metabolic alterations in lung epithelial cells caused by radiation, we first examined the intracellular levels of ATP and NADP(H) redox couples, which are involved in cellular energy metabolism and the maintenance of redox balance. We found that ATP levels increased, and the NADPH/NADP(H)_total_ ratio decreased after radiation in BEAS-2B cells (Fig. 3D). We then investigated the glycolysis level after radiation at different time points and found that the lactic acid level in supernatants was significantly increased after radiation, while the glucose concentration was decreased, suggesting upregulated glycolysis in irradiated epithelial cells (Fig. 3E).

Studies have shown that p53 plays a vital role in antioxidant response and cell metabolism [27]. GSEA revealed that the p53 pathway and glycolysis were enriched in irradiated BEAS-2B cells (Fig. 3C). Among the core enriched genes, the most significant differentially expressed gene was dihydropyrimidinase-like 4 (DPYSL4) (Fig. 3C), which has been reported to be p53-inducible and is associated with OXPHOS and cellular energy supply [28]. Therefore, we examined the expression levels of DPYSL4 after radiation. We found that DPYSL4 was significantly upregulated in BEAS-2B and PHBE cells post-irradiation (Fig. 3F). Next, we sought to determine the function of DPYSL4 in irradiated epithelial cells. DPYSL4 silencing resulted in a lower ATP level and a higher NADPH/NADP(H)_total_ ratio than in the control group after radiation (Fig. 3F, G). Since NADP^+^/NADPH redox couples are essential for maintaining cellular redox homeostasis [29], we examined the cellular ROS levels in DPYSL4-silencing or small interfering control lung epithelial cells. We found that silencing DPYSL4 greatly increased cellular ROS levels either in the presence or absence of radiation (Fig. 3I), suggesting a protective effect of DPYSL4 post radiation by regulating cellular energy metabolism. Consistent with the above findings, silencing DPYSL4 increased IL-1β secretion in BEAS-2B and PHBE cells, possibly due to increased cellular ROS levels (Fig. 3H).

To explore the role of ROS in RILI, the ROS levels in irradiated lung tissue of mice were detected. The results showed that the ROS level was significantly increased at day 7 post radiation and remained at high level until 5 months after radiation compared with that of unirradiated mice, indicating that ROS was involved in the whole process of RILI (Additional file 1: Fig. S6A). Moreover, immunohistochemistry revealed that DPYSL4 was greatly upregulated in bronchus 7 days after radiation and continued until 5 months after radiation (Additional file 1: Fig. S6B).

### NLRP3 inflammasome activation triggers fibroblast mature and migration through IL-1β

**Fig. 4:**
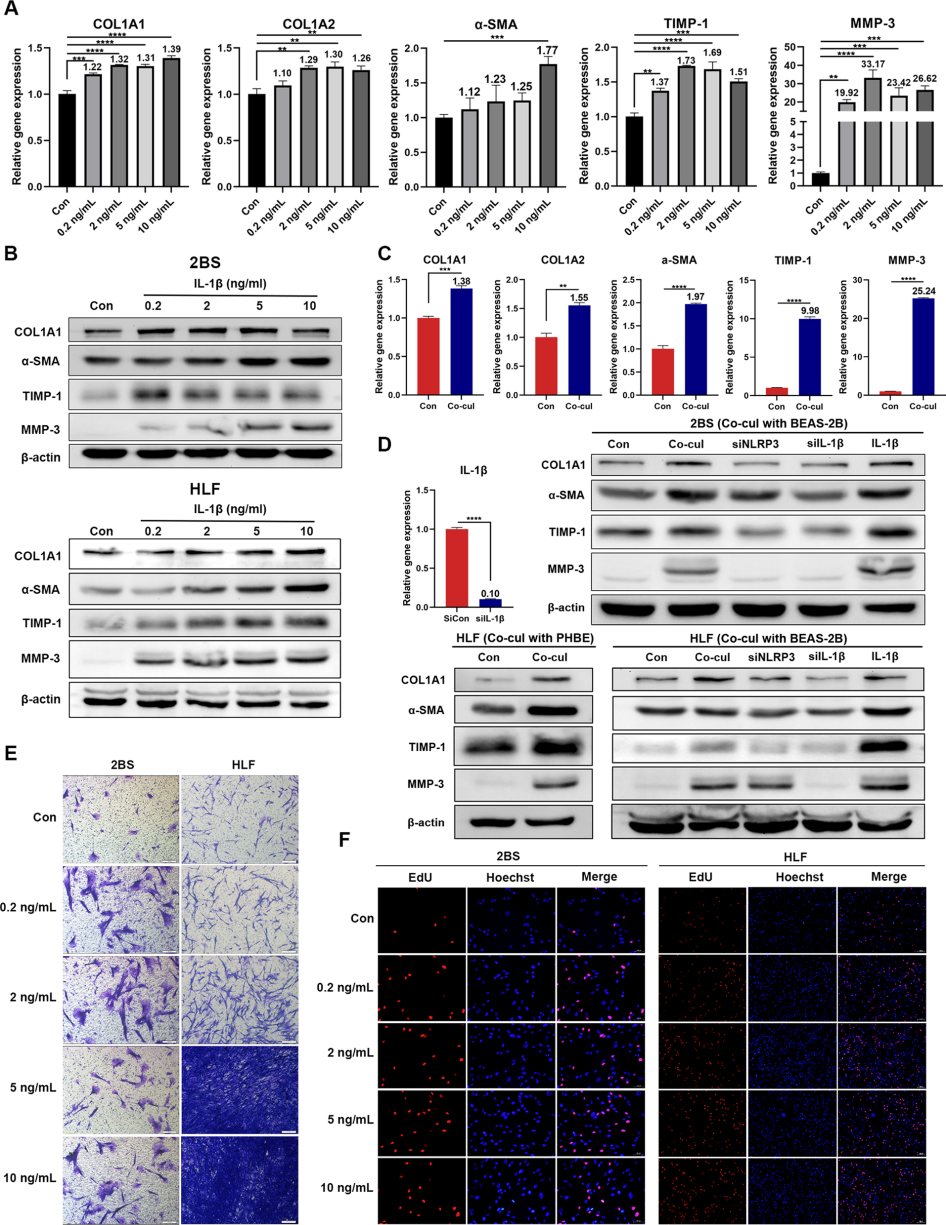
IL-1β promotes the proliferation, migration, and activation of fibroblasts. **A** qRT-PCR showing mRNA levels of COL1A1, COL1A2, α-SMA, TIMP-1, and MMP-3 in 2BS after IL-1β treatment for 24 h (n = 3). **B** Western blot showing the protein levels of COL1A1, α-SMA, TIMP-1, and MMP-3 in 2BS and HLF after IL-1β treatment for 24 h (n = 3). **C** qRT-PCR showing the mRNA levels of COL1A1, COL1A2, α-SMA, TIMP-1, and MMP-3 in 2BS after co-culture with conditioned medium from irradiated BEAS-2B for 24 h (n = 3). **D** BEAS-2B cells were transfected with control or IL-1β siRNA and analyzed by qRT-PCR to confirm knockdown efficiency (n = 3). Western blotting was performed to examine the protein levels of COL1A1, α-SMA, TIMP-1, and MMP-3 in 2BS and HLF after co-culture with conditioned medium of irradiated BEAS-2B cells transfected with control, NLRP3, or IL-1β siRNA for 24 h, and the protein levels of COL1A1, α-SMA, TIMP-1, and MMP-3 in HLF after co-culture with conditioned medium of irradiated PHBE (n = 3). **E** Representative microscopy images of the transwell assay showing that IL-1β promoted the migration of 2BS and HLF (n = 3). **F** EdU assay showing that IL-1β promotes the proliferation of 2BS and HLF (n = 3). Bar graphs show the mean ± SEM; **P < 0.01, ***P < 0.001, ****P < 0.0001. *IL* interleukin, *qRT-PCR* quantitative reverse transcription polymerase chain reaction, *TIMP* tissue inhibitors of metalloproteinases, *MMP* matrix metalloproteinases, *siRNA* small interfering RNA, *HLF* primary human fibroblast, *PHBE* primary human bronchial epithelial cells, *SEM* standard error of the mean

A broad spectrum of molecules, including extracellular matrix (ECM) components, collagens, and ECM modulators, matrix metalloproteinases (MMPs), and tissue inhibitors of metalloproteinases (TIMPs), have been reported to participate in the process of lung fibrosis. To determine the effect of IL-1β on RPF, the human fibroblast cell line, 2BS, was used. qRT-PCR and western blotting were performed to observe the expression of collagens, TIMPs, and MMPs, as well as the fibroblast activation marker α-SMA. The results showed that IL-1β induced the expression of type I collagens, TIMP-1, MMP-3, and α-SMA in fibroblasts (Fig. 4A, B), indicating the effect of IL-1β on fibroblast activation and lung tissue remodeling. To validate these results, primary human lung fibroblasts (HLF) and mouse embryonic fibroblasts (MEF) were extracted (Additional file 1: Fig. S7A). Consistent with the results obtained from cell line 2BS, we found that IL-1β promoted the synthesis of type I collagen, α-SMA, TIMP-1, and MMP-3 in HLF and MEF (Fig. 4B, Additional file 1: Fig. S7B). To ascertain that the epithelium-derived IL-1β was responsible for the observed phenotype change, the culture medium of BEAS-2B was collected after 48 h of incubation post radiation, then added to 2BS and co-cultured for 24 h. Similar to the treatment with IL-1β, the conditioned medium significantly induced the mRNA expression of COL1A1, COL1A2, TIMP-1, MMP-3, and α-SMA (Fig. 4C). To further confirm whether NLRP3 inflammasome activation in irradiated lung epithelial cells mediated the response of fibroblasts, BEAS-2B cells were transfected with NLRP3, IL-1β siRNA, or control siRNA and then treated with 10 Gy radiation. The conditioned medium was collected 48 h later and co-cultured with 2BS or HLF. Both silencing NLRP3 and IL-1β in BEAS-2B cells significantly suppressed the activation of fibroblasts, and silencing IL-1β eliminated the effect of co-culturing, indicating that other pathways that activate IL-1β may be involved in fibroblast activation. If IL-1β was added to the conditioned medium of irradiated IL-1β-silencing BEAS-2B, the activation effect was restored (Fig. 4D). Similarly, the conditioned medium of irradiated PHBE greatly promoted the activation of HLF, further confirming the above results (Fig. 4D).

Next, we investigated whether IL-1β affected the migration and proliferation of fibroblasts. Transwell assays demonstrated that IL-1β facilitated the migration ability of 2BS, HLF, and MEF (Fig. 4E, Additional file 1: Fig. S7C). The EdU experiment showed that IL-1β stimulated the proliferation of 2BS, HLF, and MEF (Fig. 4F, Additional file 1: Fig. S7D). Taken together, our results suggest that lung epithelial cells activate the NLRP3/IL-1β pathway after irradiation and induce the activation, proliferation, and migration of fibroblasts.

### NLRP3 inflammasome was activated and associated with RPF in vivo

**Fig. 5:**
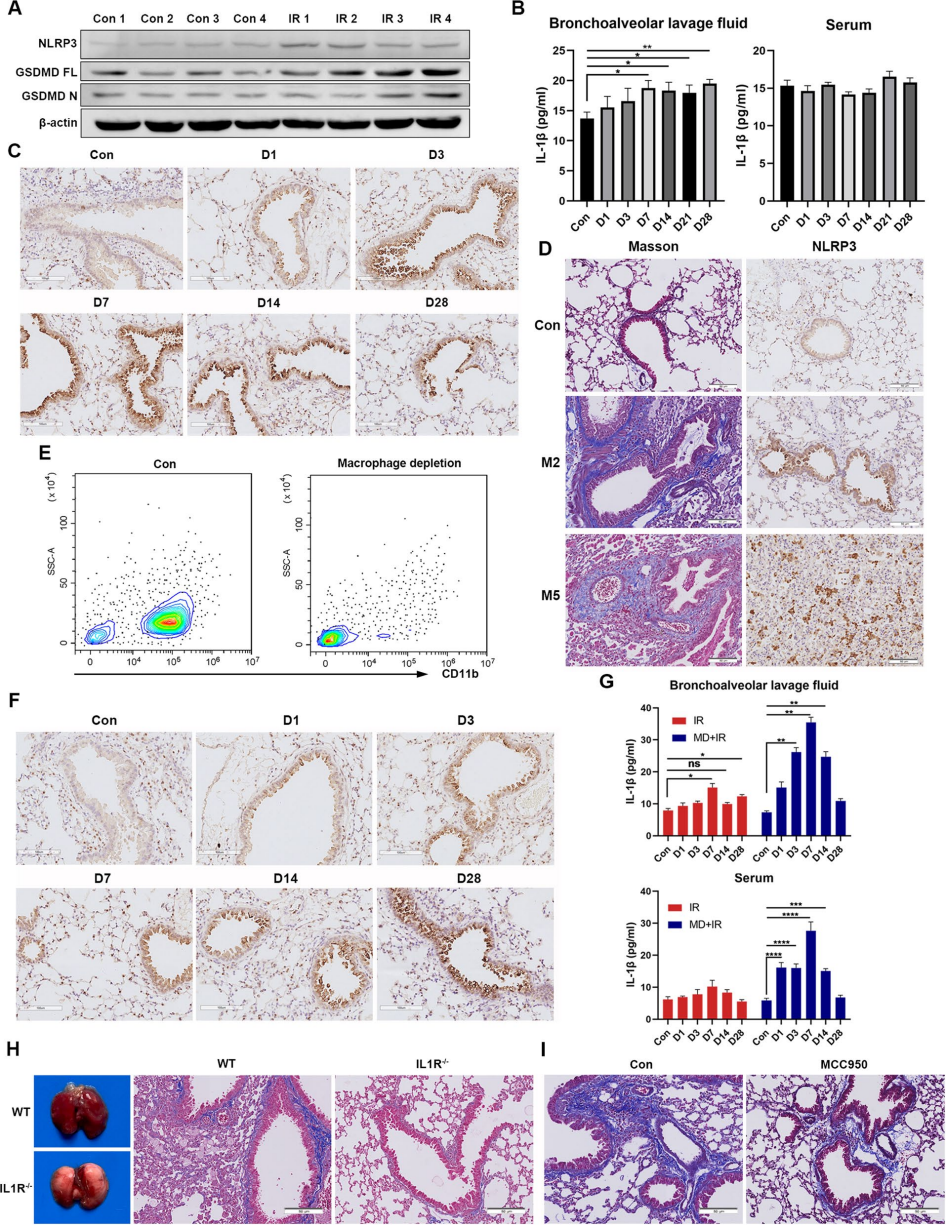
NLRP3 inflammasome is activated and promoted the progression of RPF in vivo. **A** Western blotting showing that NLRP3 protein level and GSDMD cleavage is increased in lung tissue of mice 7 days after 16 Gy thoracic X-ray radiation (n = 4). **B** ELISA results of IL-1β in BALF and serum of mice at different time points after radiation (n = 5). **C** Representative images of IHC staining for NLRP3 in lung tissue of mice at different time points after radiation (n = 5). **D** Representative images of NLRP3 and Masson’s staining in lung tissue of mice 2 and 5 months after radiation (n = 5). **E** Flow cytometry of the expression levels of CD11b in lung tissue of mice to confirm the depletion efficiency of macrophage (n = 5). **F** Representative images of IHC staining for NLRP3 in lung tissue of mice receiving macrophage depletion at different time point after radiation (n = 5). **G** ELISA results of IL-1β in BALF and serum of mice in different groups at different time points after radiation (n = 5). **H** Representative images of lung tissue and Masson’s staining of lung tissue of wild-type and IL-1R^−/−^ mice (n = 5). **I** Representative images of Masson’s staining in lung tissue of mice treated with or without MCC950 5 months after radiation (n = 5). Bar graphs show the mean ± SEM; *P < 0.05, **P < 0.01, ***P < 0.001, ****P < 0.0001. *RPF* radiation-induced pulmonary fibrosis, *ELISA* enzyme-linked immunosorbent assay, *IL* interleukin, *BALF* bronchoalveolar lavage fluid, *IHC* immunohistochemical, *WT* wild type, *1L-1R* IL-1 receptor, *SEM* standard error of mean, *ns* not significant

To further analyze the effect of the NLRP3 inflammasome in RILI, 6-week female C57Bl/6 mice were administered a single thoracic 16 Gy radiation dose to construct RILI mouse models. We evaluated the expression of the NLRP3 inflammasome in lung tissue 7 days after radiation. Radiation triggered an increase in NLRP3 and GSDMD expression in irradiated lung tissue, as revealed by western blotting (Fig. 5A). BALF and lung tissues were obtained at different time points after radiation. The IL-1β levels in BALF were significantly increased on day 7, which persisted over 21 days, while the change in IL-1β levels in serum did not reach statistical significance (Fig. 5B). IHC analysis of the lung tissue showed that NLRP3 was significantly upregulated in mouse bronchial tissue on days 1, 3, 7, 14, and 28 after radiation (Fig. 5C). Two months after radiation, Masson’s staining revealed lung tissue remodeling and collagen deposition around the bronchus, which was more evident 5 months after radiation. Furthermore, NLRP3 immunohistology remained positive 5 months post-radiation, indicating that it may participate in the whole process of RILI (Fig. 5D).

Clodronate liposomes were used to deplete the lung macrophages of mice to rule out the effect of macrophages. The depletion efficiency was confirmed by flow cytometry (Fig. 5E), and the mice were administered 16 Gy thoracic radiation. IHC analysis of the lung tissue demonstrated that NLRP3 was still positive in the bronchus after macrophage depletion (Fig. 5F). We found that IL-1β levels in the BALF of macrophage-depleted mice were significantly higher than that of control mice. Furthermore, IL-1β levels in the serum were augmented in macrophage-depleted mice, which was not observed in the mice of the control group, suggesting that macrophages may play an immunosuppressive role in the early phase of RILI (Fig. 5G). To further validate the effect of IL-1β, we used IL-1R^−/−^ mice that received 16 Gy of thoracic irradiation (Additional file 1: Fig. S8). Lung tissue was collected after 5 months. Masson’s staining revealed a significantly higher amount of collagen deposition and more severe fibrosis in wild-type mice than in IL-1R^−/−^ mice (Fig. 5H), confirming that targeting IL-1β is a promising strategy to alleviate RILI.

MCC950 has been proposed as a specific small molecule inhibitor that can selectively block NLRP3 inflammasome activation [30]. Previous study has shown that MCC950 treatment ameliorates RP and decreases cytokine production including IL-6, IL-18, and IL-1β [10]. We further explored whether MCC950 could improve RPF. Lung of irradiated mice were collected after 5 months. Masson’s staining showed that MCC950 significantly alleviated lung fibrosis induced by radiation (Fig. 5I). These findings suggested that MCC950 was a promising drug for the prevention of RPF.

**Fig. 6:**
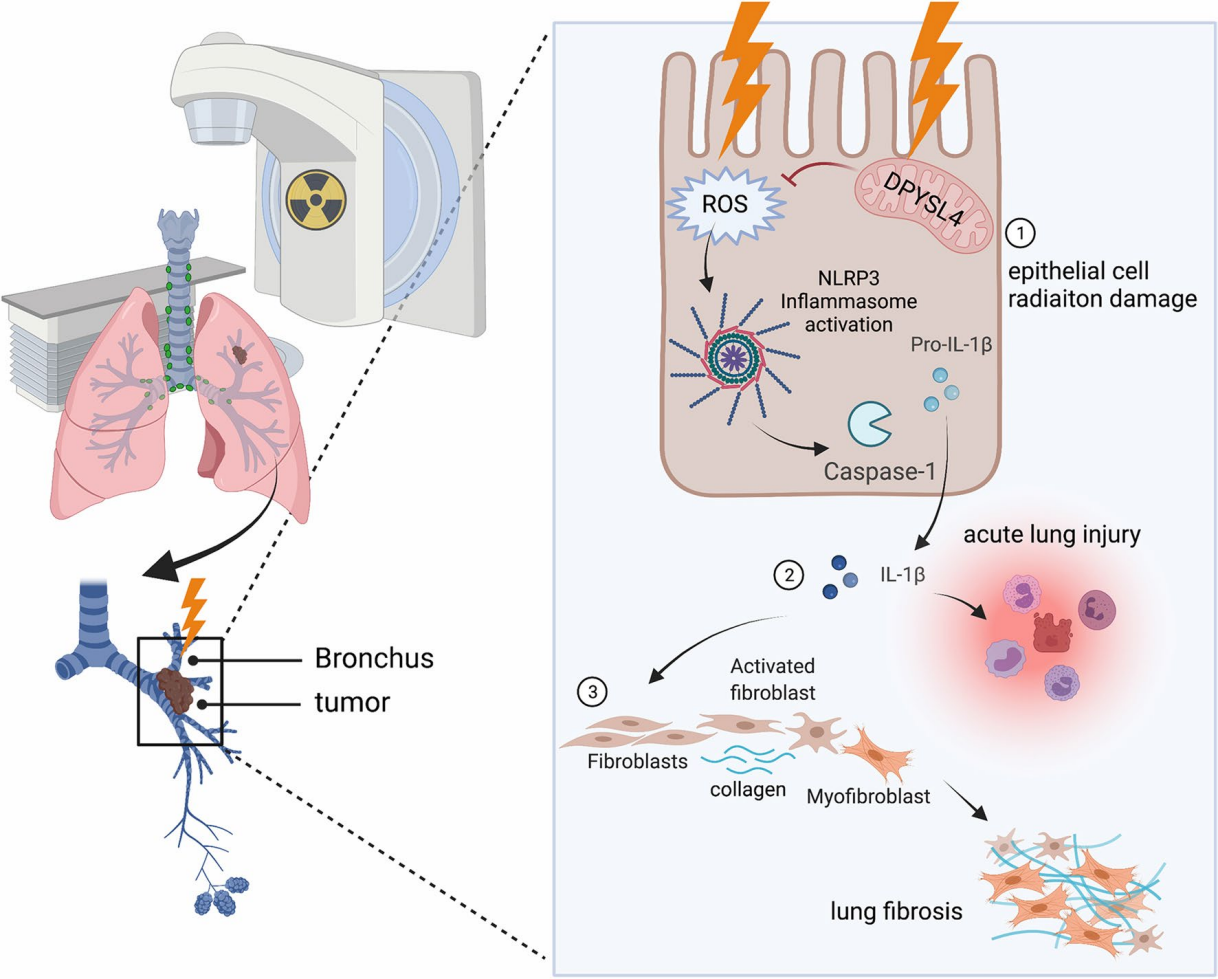
A schematic illustration showing that radiation induced NLRP3 inflammasome activation in lung epithelial cells is associated with glucose metabolic alteration involving DPYSL4 and promotes RILI progression through IL-1β secretion. *RILI* radiation-induced lung injury, *IL* interleukin

Here, we demonstrated that after radiation, the NLRP3 inflammasome is activated in lung epithelial cells, where DPYSL4 regulated glucose metabolism alteration is related to intracellular ROS accumulation and is thus associated with NLRP3 inflammasome activation. IL-1β secreted by lung epithelial cells promotes the activation, proliferation, and migration of fibroblasts as well as promotes the process of lung tissue remodeling, thereby promoting the progression of RILI (Fig. 6).

## Discussion

RILI is one of the most common fatal complications of thoracic radiotherapy [31]. Previous studies have mainly focused on how to increase the radiosensitivity of tumor cells, thus decreasing the total radiation dose to decrease radiation injury [32]. However, most molecular targets that sensitize tumor cells to radiotherapy also increase damage to the normal lung epithelium.

This study investigated the different response patterns of normal lung cells and tumor cells to radiation, thus discovering potential radioprotective drugs. We reported tremendous differences in molecular pathway changes between normal and malignant lung cells after radiation damage using RNA-seq. Despite the different activation of p53 and cell cycle pathways. Surprisingly, the inflammatory response involving NLRP3 was only activated in lung epithelial cells but not in lung cancer cells after radiation. We found that NLRP3 is the highest-ranked inflammasome and its canonical pathway activation is associated with altered glucose metabolism. IL-1β, as the direct downstream protein of NLRP3, accelerates RILI by promoting fibroblast proliferation, migration, and activation as well as lung tissue remodeling. The repression of NLRP3 or IL-1β significantly reversed RILI in vitro and in vivo. All phenotypes were also observed in human-derived primary cells.

The tumor-killing effect of radiation can also damage the normal epithelium. The tolerable dose to normal tissues is a key issue limiting the implementation of radiotherapy. The concurrent combination of chemotherapy or immunotherapy and radiotherapy improves efficacy but also promotes the occurrence of RILI [33]. Studies have revealed that the development of RILI involves multiple processes, including DNA damage and ROS generation, inflammatory cell infiltration, hypoxia, and lung tissue remodeling [10, 31, 34]. To identify a potential protective agent against RILI, we analyzed the different response patterns of lung cancer and lung epithelial cells to radiation injury using RNA-seq. Consistent with previous studies, radiation triggers a series of DNA damage response pathways in cancer cells [35, 36], including those helping cells recover from radiation injuries, such as activation of DNA damage sensing and early transduction pathways, cell cycle arrest, and DNA repair. The epithelium tends to undergo DNA damage-induced senescence and death, and triggers an inflammatory response. These differences provide a direction for us to seek potential agents for the prevention of RILI.

The NLRP3 inflammasome is widely expressed in epithelial and immune cells [37]. It has been reported to play an important role in autoimmune and metabolic diseases by activation of CASP1 and production of IL-1β and IL-18. It is involved in the development of IPF and infective lung injury [38]. However, their role in cancer remains controversial [39]. Canonical activation of the NLRP3 inflammasome enhances the proliferation and metastasis of lung adenocarcinoma cells [40]. It has also been shown to have an anti-tumorigenic role by promoting dendritic cell-mediated priming of IFN-γ-producing T lymphocytes against tumor cells [41]. Activation of the NLRP3 inflammasome pathway has also been revealed in cancer-associated fibroblasts to functionally promote tumor progression and metastasis by modulating the tumor microenvironment towards an immunosuppressive milieu and by upregulating the expression of adhesion molecules on endothelial cells [42]. Consistent with previous findings, we found that MCC950, a specific small-molecule inhibitor of NLRP3, could significantly inhibit the proliferation of both small cell lung cancer (SCLC) and non-SCLC cells. Furthermore, it significantly alleviated lung fibrosis induced by radiation. Although the NLRP3 inflammasome was first identified and well-studied in immune cells, it can be activated by low-dose irradiation both in vitro and in vivo in macrophage [43], and inhibition or deletion of NLRP3 can specifically alleviate radiation-induced lung inflammation in radiation [10]. We reported that the NLRP3 inflammasome canonical pathway is activated in lung epithelial cells in response to radiation-induced damage, contributing to pulmonary fibrosis by activating fibroblasts. We used a macrophage scavenger to eliminate the effect of macrophages in the lungs in an in vivo mouse model of RILI. Mice eliminated with macrophages showed a higher level of IL-1β, suggesting that the role of macrophages may be protective in the early phase of RILI. The limitation here is that, since the differentiation of macrophages is heterogeneous, we did not distinguish the roles of different subtypes of macrophages. In addition, we only observed the early effects of macrophage depletion, and its role in the late phase of RILI remains unclear.

IL-1β is a classical downstream effector molecule of NLRP3, and previous studies have demonstrated the role of IL-1β in pulmonary fibrosis [8]. Here, we demonstrated that it is the actual functional protein of NLRP3 that induces fibroblast proliferation and migration. We confirmed that the activation and accumulation of fibroblasts significantly induced the synthesis of type I collagen, which is a key element in pulmonary fibrosis. Another important feature of pulmonary fibrosis is pulmonary remodeling [44]. We found that IL-1β induced TIMP-1 and MMP-3 expression, indicating the role of IL-1β in pulmonary remodeling. These data support the central role of IL-1β in RPF, making it an attractive novel therapeutic target.

We observed that early glycolysis products may be diverted to lactic acid instead of the citric acid cycle when in need of faster ATP production. Previous evidence suggests that alterations in glycolysis and glucose metabolism contribute to IPF progression [45]. The Warburg effect induces myofibroblast differentiation and contributes to fibrosis in IPF [15]. Lactic acid in the tumor microenvironment has been reported to suppress anticancer immunity [46]. Therefore, targeting glucose metabolic dysregulation and suppressing the Warburg effect may be a promising future direction for RILI. We found that DPYSL4, a p53-inducible gene, was upregulated in irradiated epithelial cells. DPYSL4 is associated with mitochondrial supercomplexes, stimulates ATP production, and suppresses cancer cell invasion [28]. Our study revealed that DPYSL4 has a protective role in irradiated epithelial cells by increasing ATP and NADPH levels and decreasing ROS levels, thus suppressing the occurrence of pyroptosis. Future research should focus on unveiling the genes and metabolites involved in glucose metabolism and their association with RILI.

Given the important role of NLRP3 and IL-1β in RILI development and progression, it is likely that repressing them using inhibitors may contribute to protecting lung function from radiation. IL-1β inhibitors, such as canakinumab, have been proven safe in lung cancer patients and have a survival benefit for certain groups [47]. The relationship between metabolic alterations and inflammasome activation provides insight into the treatment and prediction model of RILI. This may be a new direction for research on the mechanisms of radiation damage and a novel target for developing new drugs in the future.

In conclusion, our data showed that glucose metabolism-related NLRP3 inflammasome activation plays a crucial role in RILI via IL-1β. This suggests the potential clinical application of IL-1β and NLRP3 inhibitors as radioprotective agents.

## Supplementary Information

**Additional file 1: Figure S1.** NLRP3 is upregulated in HBE while remains unchanged in A549 and H446. (A) Western blotting showing the protein level of NLRP3, CASP1 cleavage and GSDMD cleavage were increased after radiation in HBE (n = 3). (B) Western blotting showing the protein level of NLRP3 is not upregulated in A549 and H446 (n = 3). **Figure S2.** ELISA results of IL-18 in supernatant of BEAS-2B, H446, A549 and H460 at different time point after radiation (n = 3). **Figure S3.** qRT-PCR showing the increase of mRNA level of NLRP3, CASP1, IL-1β and GSDMD expression after radiation were inhibited in BEAS-2B transfected with NLRP3 siRNA (n = 3). Bar graphs show the mean ± SEM; ***P < 0.001, ****P < 0.0001. SEM: standard error of mean; ns: not significant. **Figure S4.** MCC950 inhibits the proliferation of lung cancer cells. (A) CCK-8 assay was performed to measure the half-maximal inhibitory concentration (IC50) of MCC950 (n = 3). (B) MCC950 significantly decreased the colony formation ability of H446 and A549 (n = 3). (C) MCC950 inhibited the proliferation ability of H446 and A549 (n = 3). Bar graphs show the mean ± SEM; **P < 0.01. SEM: standard error of mean. **Figure S5.** Radiation induced intracellular ROS accumulation triggers NLRP3 inflammasome activation in HBE. (A) Flow cytometry of the intracellular ROS level at 6h post radiation pretreated with or without NAC in HBE (n = 3). (B) Western blotting showing the protein level of NLRP3, ASC, CASP1 cleavage and GSDMD cleavage were inhibited pretreated with NAC after radiation in HBE (n = 3). Bar graphs show the mean ± SEM. **P < 0.01; ***P < 0.001; ****P < 0.0001. ROS: reactive oxygen species; NAC: *N*-acetyl-l-cysteine; SEM: standard error of mean. **Figure S6.** The ROS and DPYSL4 levels are increased in irradiated lung tissue of mice. (A) The ROS levels were increased in RILI (n = 5). (B) Representative images of IHC staining for DPYSL4 in lung tissue after radiation (n = 5). Bar graphs show the mean ± SEM. ****P < 0.0001. ROS: reactive oxygen species; SEM: standard error of mean. **Figure S7.** IL-1β promotes the proliferation, migration, and activation of MEF. (A) Microscopy images of HLF and MEF. Scale bar, 100 μm. (B) Western blotting showing the protein level of COL1A1, α-SMA, TIMP-1 and MMP-3 in MEF after IL-1β treatment for 24h (n = 3). (C) Representative microscopy images of transwell assay showing IL-1β promoted the migration of MEF (n = 3). (D) EdU assay showing IL-1β promoted the proliferation of MEF (n = 3). HLF: primary human fibroblast; MEF: primary mouse embryonic fibroblast. **Figure S8.** Gel electrophoresis was performed to confirm the knockout of IL1R of mice (n = 3). H_2_O, no template control group. B6, C57BL/6N wild type mice. 12&13, IL1R^−/−^ mice.

**Additional file 2: Table S1.** The primer sequences used in qRT-PCR. **Table S2.** Primer sequence of IL1R mutant and wild type.

## Data Availability

The dataset(s) supporting the conclusions of this article is(are) included within the article and its Additional files.

## Abbreviations

RILI: Radiation-induced lung injury
RP: Radiation pneumonitis
RPF: Radiation-induced pulmonary fibrosis
IPF: Idiopathic pulmonary fibrosis
NLRP: Nucleotide-binding oligomerization domain-like receptor family pyrin domain-containing
ASC: An apoptosis-associated speck-like protein containing a caspase-recruitment domain
CASP1: Caspase-1
IL-1β: Interleukin -1β
DPYSL4: Dihydropyrimidinase-like 4
OXPHOS: Mitochondrial oxidative phosphorylation
PBS: Phosphate-buffered saline
ATP: Adenosine 5′-triphosphate
BALF: Bronchoalveolar lavage fluid
FBS: Fetal bovine serum
PHBE: Primary human bronchial epithelial cell
ELISA: Enzyme-linked immunosorbent assay
GSEA: Gene set enrichment analysis
ROS: Reactive oxygen species
IHC: Immunohistochemistry
HLF: Primary lung fibroblasts
MEF: Primary mouse embryo fibroblasts
GSDMD: Gasdermin D
RNA-seq: RNA-sequencing
siRNA: Small interfering RNA
SCLC: Small cell lung cancer
NADP: Nicotinamide adenine dinucleotide phosphate
NAC: *N*-Acetyl-l-cysteine
ECM: Extracellular matrix
MMPs: Matrix metalloproteinases
TIMPs: Tissue inhibitors of metalloproteinases
SEM: Standard error of mean
