# Supplementary material for: Activation of NLRP3 inflammasome in lung epithelial cells triggers radiation-induced lung injury
Source: Respir Res. 2023 Jan 24;24:25. doi: 10.1186/s12931-023-02331-7 (PMC9872296; doi:10.1186/s12931-023-02331-7)
Supplement: Supplementary file 1 — Additional file 1: Figure S1. NLRP3 is upregulated in HBE while remains unchanged in A549 and H446. (A) Western blotting showing the protein level of NLRP3, CASP1 cleavage and GSDMD cleavage were increased after radiation in HBE (n = 3). (B) Western blotting showing the protein level of NLRP3 is not upregulated in A549 and H446 (n = 3). Figure S2. ELISA results of IL-18 in supernatant of BEAS-2B, H446, A549 and H460 at different time point after radiation (n = 3). Figure S3. qRT-PCR showing the increase of mRNA level of NLRP3, CASP1, IL-1β and GSDMD expression after radiation were inhibited in BEAS-2B transfected with NLRP3 siRNA (n = 3). Bar graphs show the mean ± SEM; ***P < 0.001, ****P < 0.0001. SEM: standard error of mean; ns: not significant. Figure S4. MCC950 inhibits the proliferation of lung cancer cells. (A) CCK-8 assay was performed to measure the half-maximal inhibitory concentration (IC50) of MCC950 (n = 3). (B) MCC950 significantly decreased the colony formation ability of H446 and A549 (n = 3). (C) MCC950 inhibited the proliferation ability of H446 and A549 (n = 3). Bar graphs show the mean ± SEM; **P < 0.01. SEM: standard error of mean. Figure S5. Radiation induced intracellular ROS accumulation triggers NLRP3 inflammasome activation in HBE. (A) Flow cytometry of the intracellular ROS level at 6h post radiation pretreated with or without NAC in HBE (n = 3). (B) Western blotting showing the protein level of NLRP3, ASC, CASP1 cleavage and GSDMD cleavage were inhibited pretreated with NAC after radiation in HBE (n = 3). Bar graphs show the mean ± SEM. **P < 0.01; ***P < 0.001; ****P < 0.0001. ROS: reactive oxygen species; NAC: N-acetyl-l-cysteine; SEM: standard error of mean. Figure S6. The ROS and DPYSL4 levels are increased in irradiated lung tissue of mice. (A) The ROS levels were increased in RILI (n = 5). (B) Representative images of IHC staining for DPYSL4 in lung tissue after radiation (n = 5). Bar graphs show the mean ± SEM. ****P < 0.0001. ROS: [file 12931_2023_2331_MOESM1_ESM.docx]

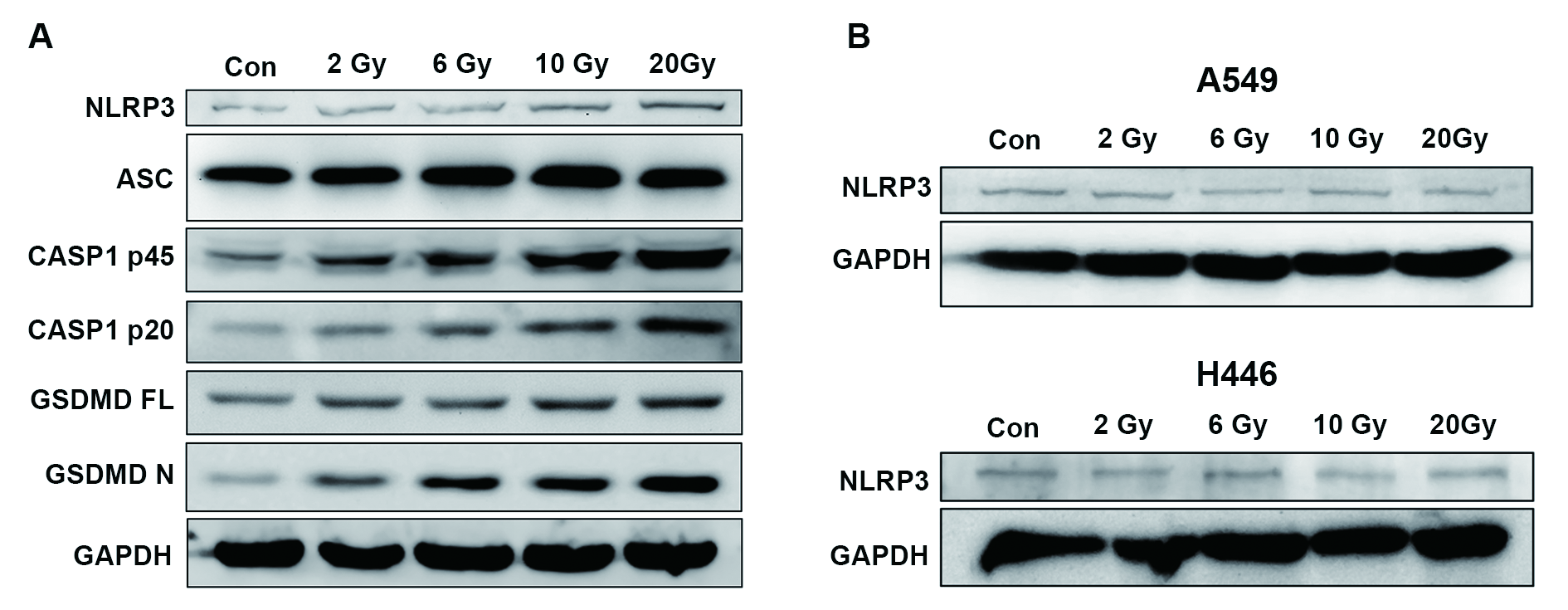


**Figure S1. NLRP3 is upregulated in HBE while remains unchanged in A549 and H446.** (A) Western blotting showing the protein level of NLRP3, CASP1 cleavage and GSDMD cleavage were increased after radiation in HBE (n=3). (B) Western blotting showing the protein level of NLRP3 was not upregulated in A549 and H446 (n=3).

**
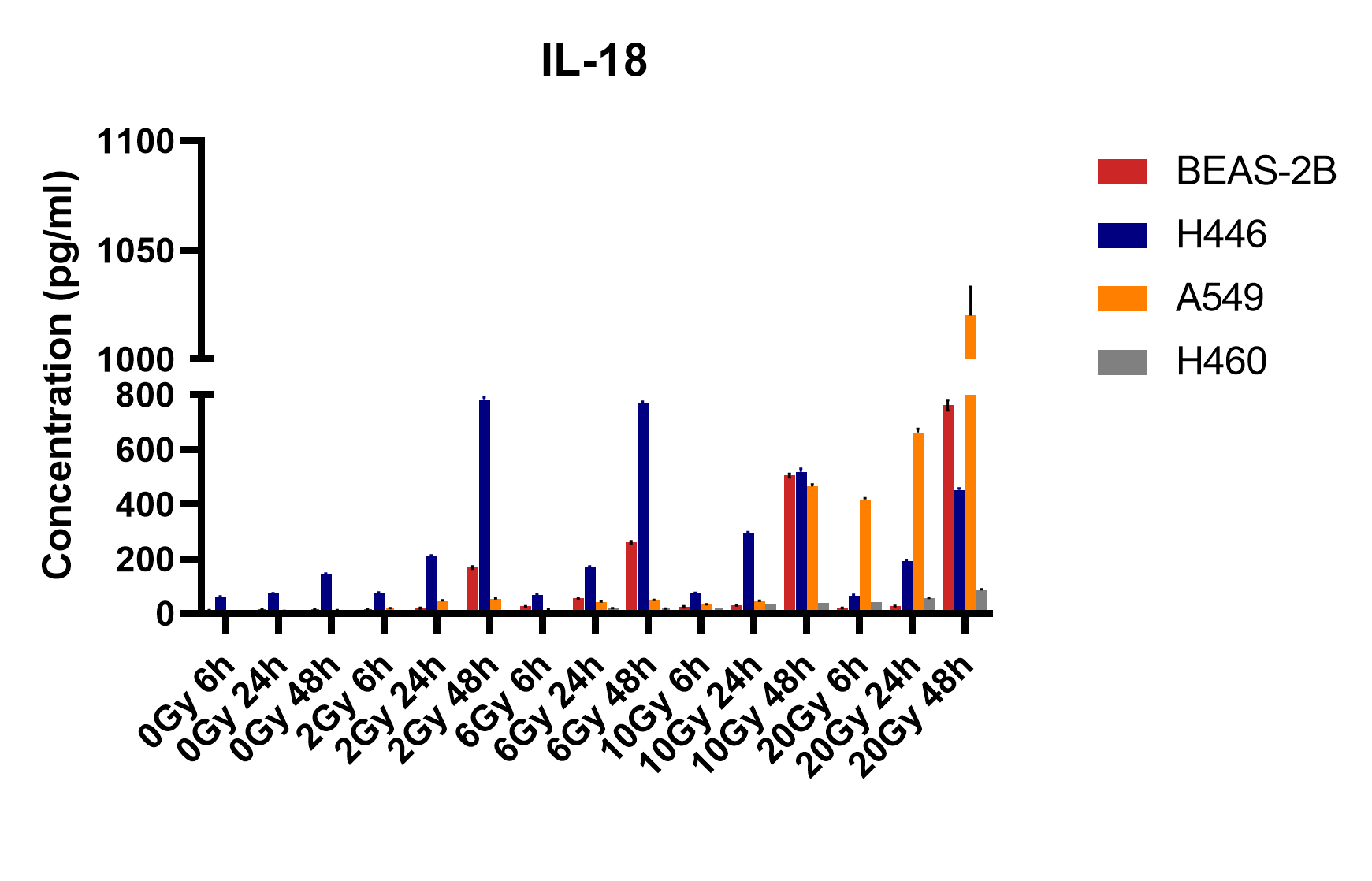
**

**Figure S2. ELISA results of IL-18 in supernatant of BEAS-2B, H446, A549 and H460 at different time point after radiation** **(n=3).**

**
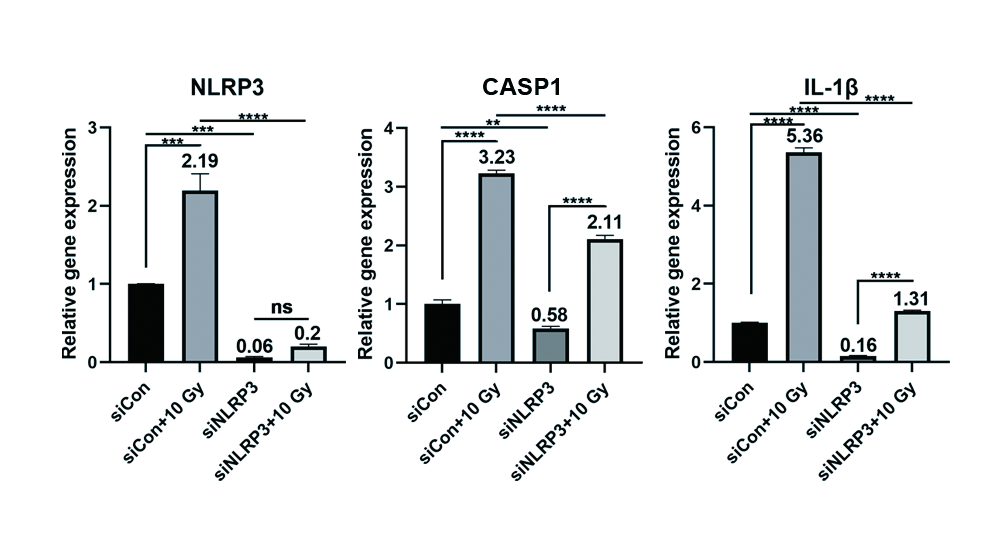
**

**Figure S3. qRT-PCR showing the increase of mRNA level of NLRP3, CASP1, IL-1β and GSDMD expression after radiation were inhibited in BEAS-2B transfected with NLRP3 siRNA (n=3).** Bar graphs show the mean ± SEM; ***P < 0.001, ****P< 0.0001. SEM: standard error of mean; ns: not significant.

**
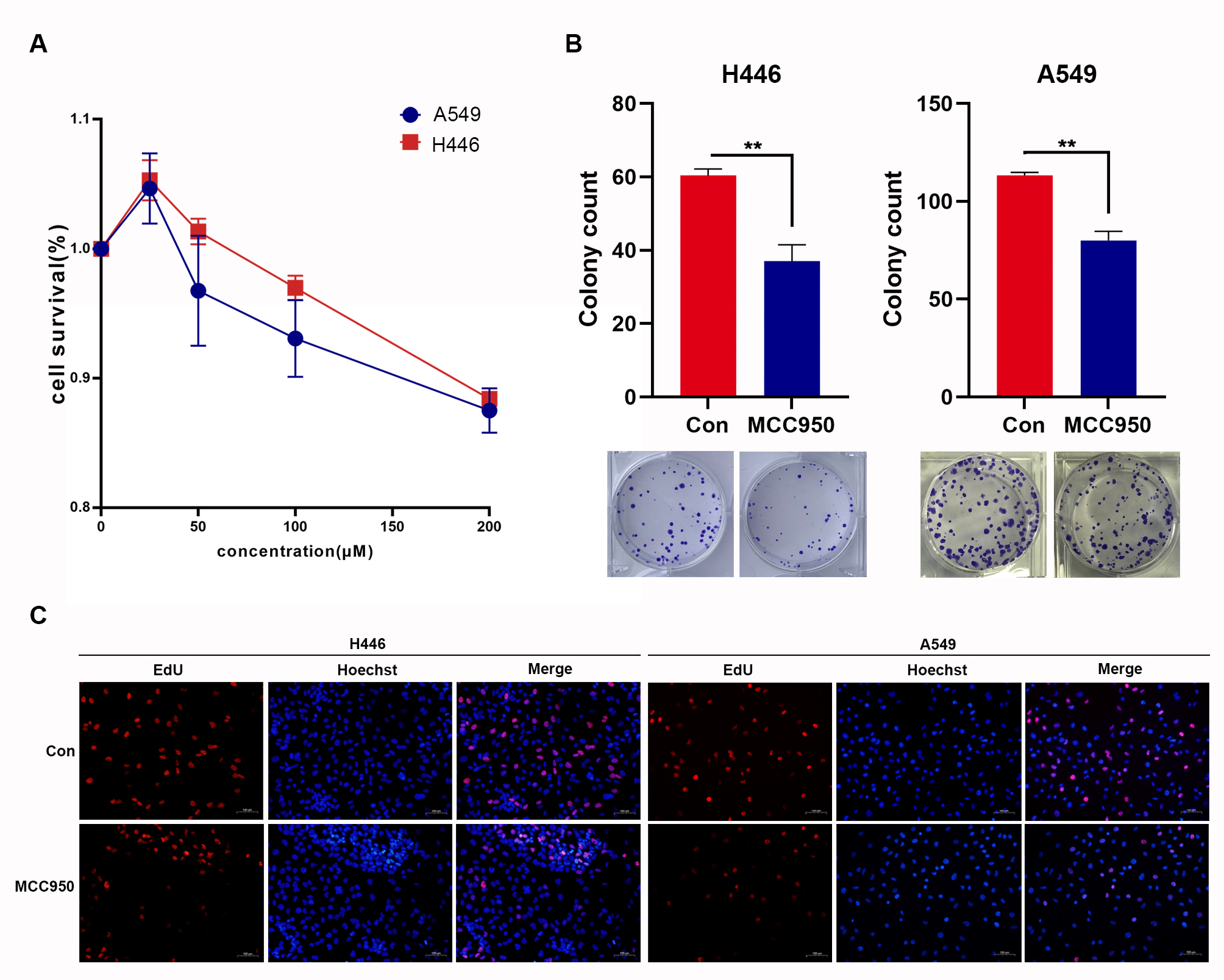
**

**Figure S4. MCC950 inhibits the proliferation of lung cancer cells.** (A) CCK-8 assay was performed to measure the half-maximal inhibitory concentration (IC50) of MCC950 (n=3). (B) MCC950 significantly decreased the colony formation ability of H446 and A549 (n=3). (C) MCC950 inhibited the proliferation ability of H446 and A549 (n=3). Bar graphs show the mean ± SEM; ** P<0.01. SEM: standard error of mean.


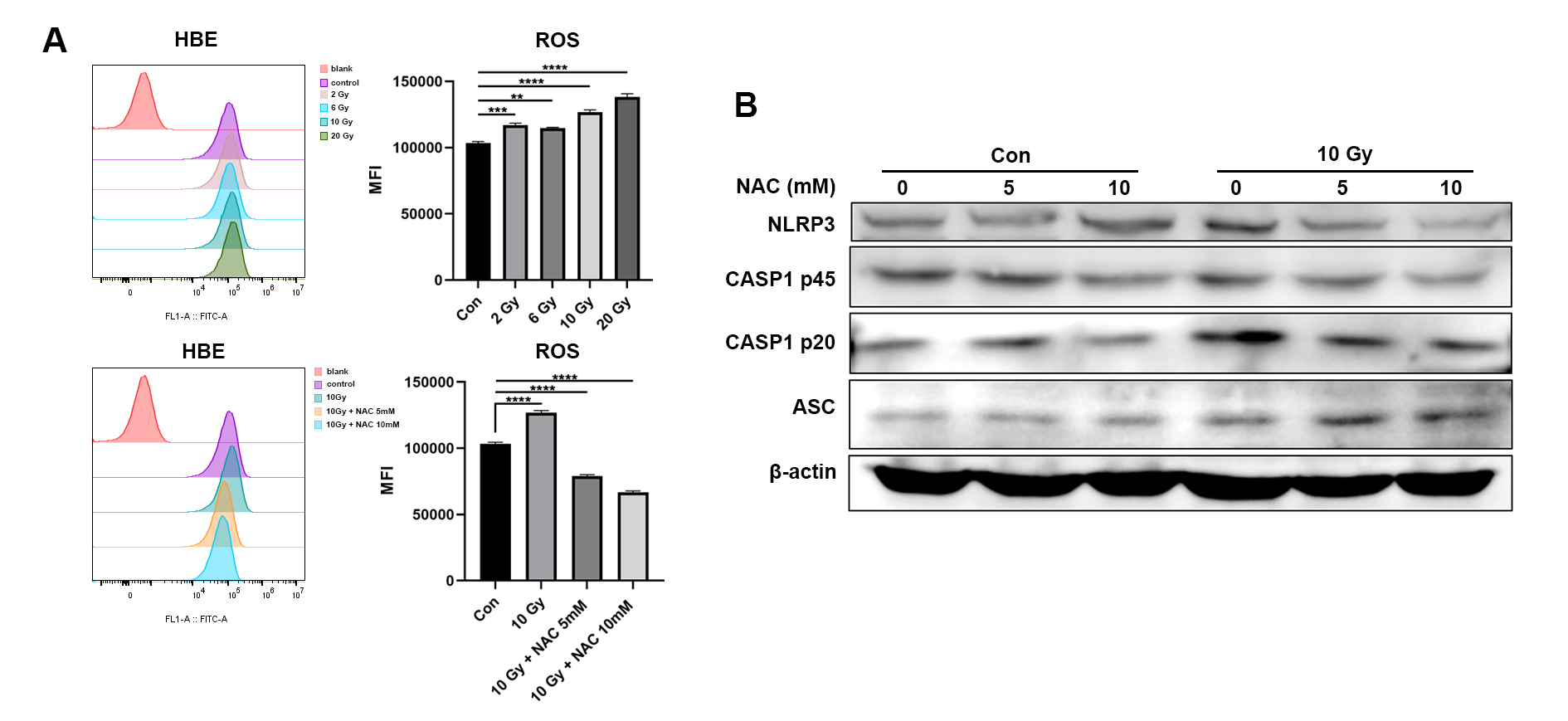


**Figure S5. Radiation induced intracellular ROS accumulation triggers NLRP3 inflammasome activation in HBE.** (A) Flow cytometry of the intracellular ROS level at 6h post radiation pretreated with or without NAC in HBE (n=3). (B) Western blotting showing the protein level of NLRP3, ASC, CASP1 cleavage and GSDMD cleavage were inhibited pretreated with NAC after radiation in HBE (n=3). Bar graphs show the mean ± SEM. ** P<0.01.*** P<0.001. **** P<0.0001. ROS: reactive oxygen species; NAC: N-acetyl-Lcysteine; SEM: standard error of mean.

**
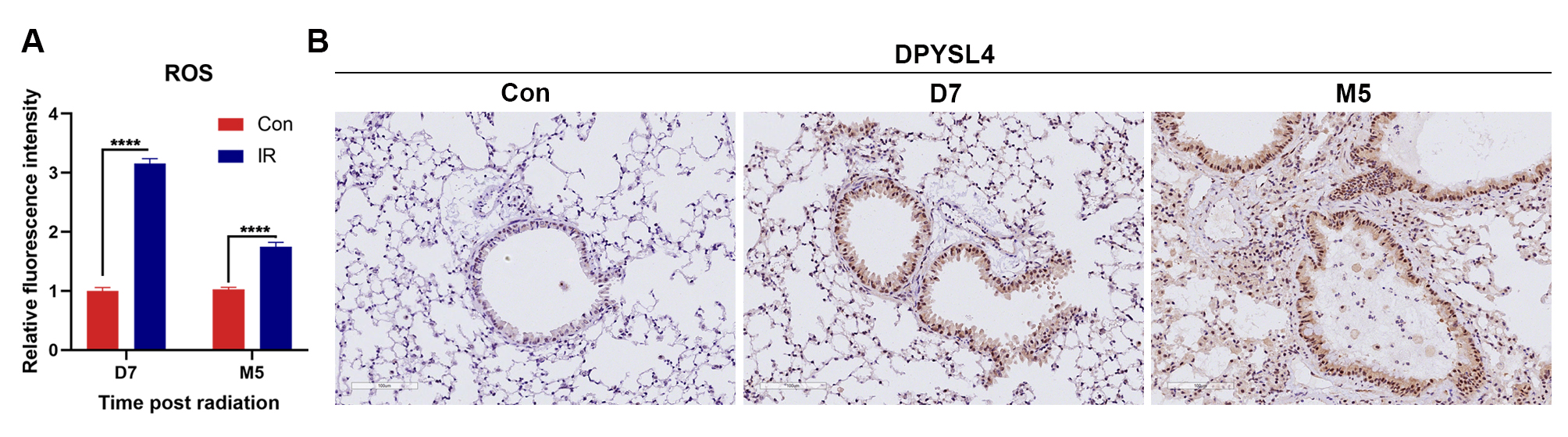
**

**Figure S6. The ROS and DPYSL4 levels are increased in irradiated lung tissue of mice.** (A) The ROS levels were increased in RILI (n=5). (B) Representative images of IHC staining for DPYSL4 in lung tissue after radiation (n=5). Bar graphs show the mean ± SEM. **** P<0.0001. ROS: reactive oxygen species; SEM: standard error of mean.


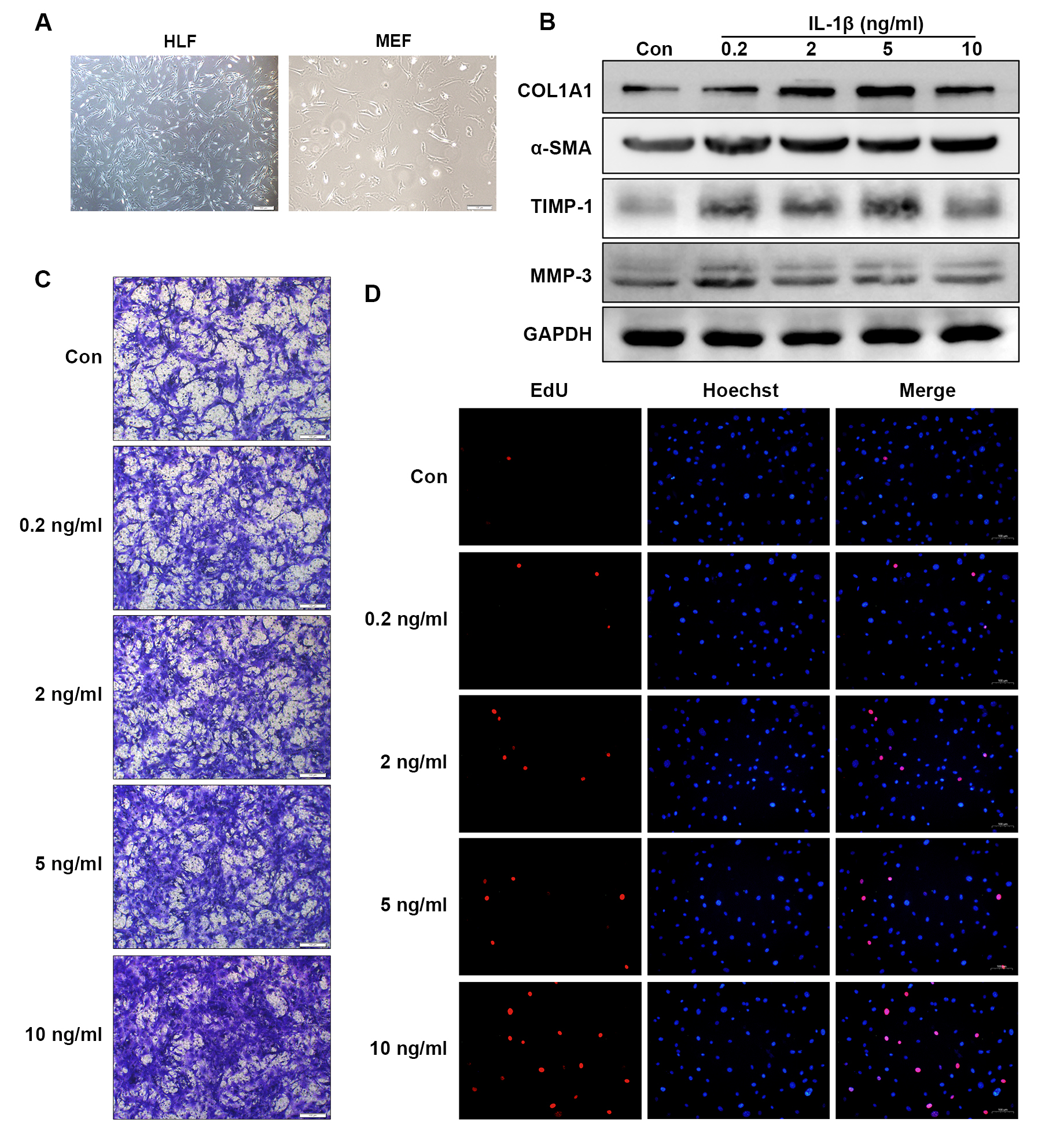


**Figure S7. IL-1β promotes the proliferation, migration, and activation of MEF.** (A) Microscopy images of HLF and MEF. Scale bar, 100 μm. (B) Western blotting showing the protein level of COL1A1, α-SMA, TIMP-1 and MMP-3 in MEF after IL-1β treatment for 24h (n=3). (C) Representative microscopy images of transwell assay showing IL-1β promoted the migration of MEF (n=3). (D) EdU assay showing IL-1β promoted the proliferation of MEF (n=3). HLF: primary human fibroblast; MEF: primary mouse embryonic fibroblast.


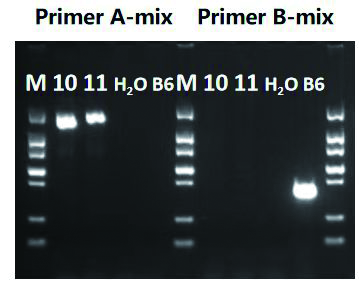


**Figure S8. Gel electrophoresis was performed to confirm the knockout of** **IL1R of mice (n=3).** H_2_O, no template control group. B6, C57BL/6N wild type mice. 12&13, IL1R^-/-^ mice.
