# Supplementary material for: Activation of NLRP3 inflammasome in lung epithelial cells triggers radiation-induced lung injury
Source: Respir Res. 2023 Jan 24;24:25. doi: 10.1186/s12931-023-02331-7 (PMC9872296; doi:10.1186/s12931-023-02331-7)
Supplement: Supplementary file 2 — Additional file 2: Table S1. The primer sequences used in qRT-PCR. Table S2. Primer sequence of IL1R mutant and wild type. [file 12931_2023_2331_MOESM2_ESM.docx]

**Table S1. The primer sequences used in qRT-PCR**

| **Gene** | **Forward 5’ to 3’** | **Reverse 3’ to 5’** |
| --- | --- | --- |
| **DPYSL4** | CACCAACGGCATTGAGGAG | ACCGCGACGAACTCATTCTC |
| **NLRP3** | GATCTTCGCTGCGATCAACAG | CGTGCATTATCTGAACCCCAC |
| **Caspase 1** | TTTCCGCAAGGTTCGATTTTCA | GGCATCTGCGCTCTACCATC |
| **IL-1β** | ATGATGGCTTATTACAGTGGCAA | GTCGGAGATTCGTAGCTGGA |
| **GSDMD** | GTGTGTCAACCTGTCTATCAAGG | CATGGCATCGTAGAAGTGGAAG |
| **COL1A1** | GAGGGCCAAGACGAAGACATC | CAGATCACGTCATCGCACAAC |
| **COL1A2** | GTTGCTGCTTGCAGTAACCTT | AGGGCCAAGTCCAACTCCTT |
| **TIMP-1** | CTTCTGCAATTCCGACCTCGT | ACGCTGGTATAAGGTGGTCTG |
| **MMP-3** | AGTCTTCCAATCCTACTGTTGCT | TCCCCGTCACCTCCAATCC |
| **α-SMA** | AAAAGACAGCTACGTGGGTGA | GCCATGTTCTATCGGGTACTTC |
| **GAPDH** | ACCACAGTCCATGCCATCAC | TCCACCACCCTGTTGCTGTA |

**Table S2. Primer sequence of IL1R mutant and wild type**

| **IL1R** | **Forward 5’ to 3’** | **Reverse 3’ to 5’** |
| --- | --- | --- |
| Mutant | CTTGGGTGGAGGCTATTC | GAGGCGTATGCCACAAAGA |
| Wild type | GGTTTGAATGTTGGGGTTTG | CACCACCACCTGGCTCTTT |
